# Tumourigenicity and Immunogenicity of Induced Neural Stem Cell Grafts Versus Induced Pluripotent Stem Cell Grafts in Syngeneic Mouse Brain

**DOI:** 10.1038/srep29955

**Published:** 2016-07-15

**Authors:** Mou Gao, Hui Yao, Qin Dong, Hongtian Zhang, Zhijun Yang, Yang Yang, Jianwei Zhu, Minhui Xu, Ruxiang Xu

**Affiliations:** 1 Department of Neurosurgery, Daping Hospital, Third Military Medical University, Chongqing 400042, China; 2 Affiliated Bayi Brain hospital, General Hospital of PLA Army, Beijing 100700, China; 3 Department of Neurology, Fu Xing Hospital, Capital Medical University, Beijing 100038, China

## Abstract

Along with the development of stem cell-based therapies for central nervous system (CNS) disease, the safety of stem cell grafts in the CNS, such as induced pluripotent stem cells (iPSCs) and induced neural stem cells (iNSCs), should be of primary concern. To provide scientific basis for evaluating the safety of these stem cells, we determined their tumourigenicity and immunogenicity in syngeneic mouse brain. Both iPSCs and embryonic stem cells (ESCs) were able to form tumours in the mouse brain, leading to tissue destruction along with immune cell infiltration. In contrast, no evidence of tumour formation, brain injury or immune rejection was observed with iNSCs, neural stem cells (NSCs) or mesenchymal stem cells (MSCs). With the help of gene ontology (GO) enrichment analysis, we detected significantly elevated levels of chemokines in the brain tissue and serum of mice that developed tumours after ESC or iPSC transplantation. Moreover, we also investigated the interactions between chemokines and NF-κB signalling and found that NF-κB activation was positively correlated with the constantly rising levels of chemokines, and vice versa. In short, iNSC grafts, which lacked any resulting tumourigenicity or immunogenicity, are safer than iPSC grafts.

With advances in regenerative medicine and reprogramming technology, stem cell-based therapies offer more hope for human beings than ever before^1^. Nevertheless, we must be mindful of the potential risks and challenges. Therefore, the safety of stem cell grafts has always been the primary problem in experimental and clinical research^2^. Studying the safety of stem cell-based therapies is a very complicated endeavour that involves many issues, with the tumourigenicity and immunogenicity of stem cell grafts being among the most important safety metrics for stem cell-based therapies^3^,^4^.

With their capacity for self-renewal and multipotency, embryonic stem cells (ESCs) and induced pluripotent stem cells (iPSCs) have the potential to form tumours^5^. In addition, iPSCs generated from autologous somatic cells via reprogramming, as well as allogeneic ESCs, may be rejected by the host immune system^6^. For instance, some scientists have reported that iPSC grafts failed to form teratoma in syngeneic mice because the immunogenicity of iPSCs led to massive immune cell infiltration^7^. Thus, the tumourigenicity and immunogenicity of stem cell grafts are tightly linked. Furthermore, whether reprogramming technology contributes to the immunogenicity of iPSCs and whether iPSC grafts can survive and form tumours in normal syngeneic mice remain controversial^8^,^9^,^10^.

In contrast to ESCs and iPSCs, neural stem cells (NSCs) and mesenchymal stem cells (MSCs), which have limited potential for proliferation and differentiation, can hardly form tumours *in vivo*^3^,^4^. Moreover, major histocompatibility complex (MHC)-class I and II molecules, as well as some antigens, are weakly expressed in NSCs and MSCs^11^,^12^. NSC- and MSC-based therapies may therefore be safer. In addition, induced neural stem cells (iNSCs) directly generated from autologous somatic cells are more suitable for regenerative medicine because they have similar capacity self-renewal and differentiation as NSCs and are more convenient than NSCs, without any fears of ethical concerns or limited sources. Recent researches have suggested that defined sets of transcription factors such as (Brn4/Pou3f4, Sox2, Klf4, c-Myc, plus E47/Tcf3)^13^, (Brn4, Sox2, Klf4, plus c-Myc)^14^,^15^, (Sox2, Klf4, c-Myc, plus Oct4)^16^, and even a single factor (Sox2)^17^ can directly reprogram mouse embryonic fibroblasts (MEFs) to stably expandable iNSCs without passing through a pluripotent state.

We previously reported that iNSCs directly reprogrammed from MEFs by conditional overexpressing (Oct4, Sox2, Klf4, plus c-Myc) could expand and differentiate into neurons, astrocytes and oligodendrocytes^18^. Moreover, we had found that iNSC grafts gave rise to GFAP-positive astrocytes and Tuj1-positive neural cells in the brain of middle cerebral artery occlusion (MCAO) animals. In theory, iNSCs, like NSCs, pose a lower risk of tumour formation than ESCs and iPSCs^3^,^14^,^18^,^19^. However, reprogramming technology may cause genetic or epigenetic instability in iNSCs as well as iPSCs^20^. Therefore, whether reprogramming technology increases the propensity for tumour formation and immune rejection in iNSC-based therapy remains uncertain.

To provide a scientific basis for evaluating the safety of ESC-, iPSC-, NSC-, iNSC- and MSC-based therapies, we detected the tumourigenicity and immunogenicity of these stem cell grafts in syngeneic mouse brains. We found that ESC and iPSC grafts produced tumours, which led to brain injury along with immune cell infiltration. In contrast, no evidence of tumour formation, brain injury or immune rejection was observed in the NSC, iNSC and MSC groups. We subsequently explored the relationship between tumour formation and immune cell infiltration. Significantly elevated levels of chemokines were found in the brain tissue and serum of mice that developed tumours after ESC or iPSC transplantation. Moreover, we also examined the interactions between chemokines and NF-κB signalling and speculated that the activation of NF-κB leads to the constant increase in chemokine levels.

## Results

### Identification and characterization of stem cells

The stable and high-level expression of GFP in ESCs, iPSCs and iNSCs was observed via confocal laser scanning microscopy (CLSM). Immunofluorescence staining indicated that ESCs and iPSCs were positive for Nanog; NSCs and iNSCs were positive for Nestin. Moreover, NSCs also endogenously expressed Sox2 (Supplementary Figure S1). MSCs were analyzed using flow cytometry, which showed that these cells were positive for expression of the MSC surface markers CD29, CD44 and CD71 (>80%), while only showing low expression of haematopoietic markers CD14, CD34 and CD45 (<5%) (Supplementary Figure S2).

### Tumour formation in the brain after stem cell transplantation

**Figure 1:**
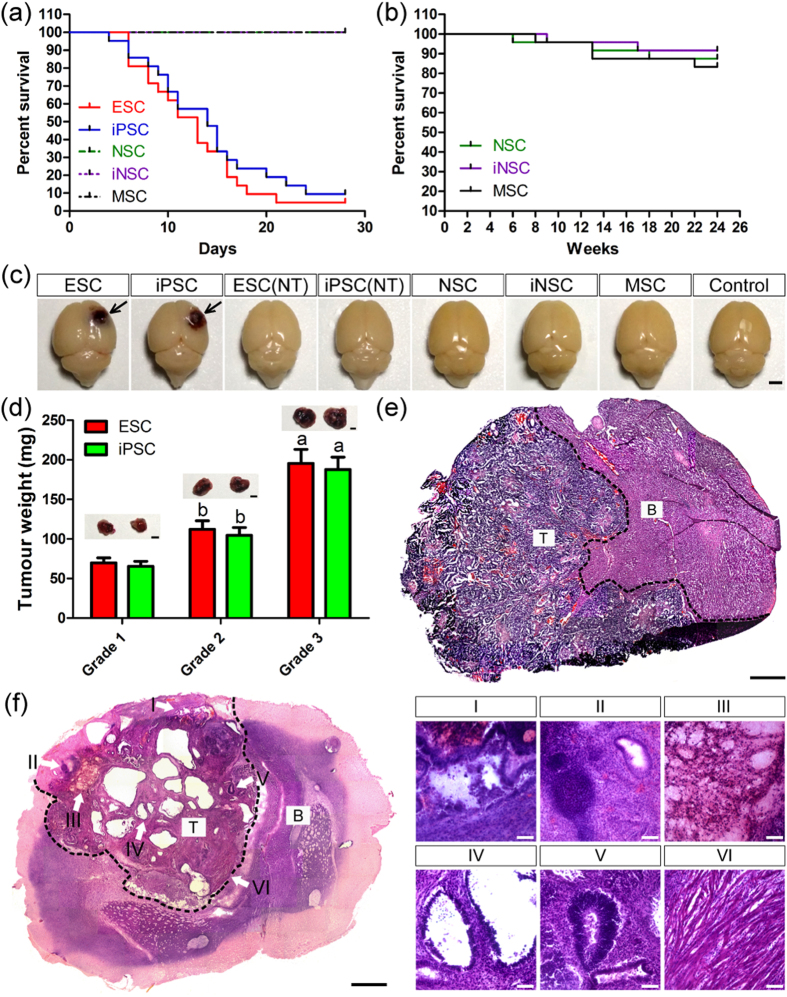
Tumour formation in the brain after stem cell transplantation. (**a**) The percent survival of animals in the ESC, iPSC, NSC, iNSC and MSC groups (n = 21/group) during the 28-day observation period. (**b**) The percent survival of animals in the NSC, iNSC and MSC groups (n = 21/group) during the 24-week observation period. (**c)** The gross morphology of brains in the ESC, iPSC, NSC, iNSC, MSC and Control groups on day 14 post-implantation (NT: no tumour formation). (**d**) Weight and representative pictures of brain tumours in the ESC (G1), iPSC (G1), ESC (G2), iPSC (G2), ESC (G3) and iPSC (G3) groups on day 14 post-implantation (n = 6/group; (**a**) *P* < 0.05 versus grade 2 or grade 1; (**b**) *P* < 0.05 versus grade 1). (**e**) Malignant teratoma formation correlated with severe brain injury and massive immune cell infiltration was detected in the brains of mice subjected to syngeneic ESC grafts on day 14 post-implantation ((B) brain tissue; T: tumour tissue). (**f)** Benign teratoma formation, including (I) cartilage tissue, (II) adenoid tissue, (III) sebaceous gland, (IV) bronchial epithelium tissue, (V) neural tissue and (VI) muscle tissue, was observed in the brains of mice subjected to syngeneic iPSC grafts on day 14 post-implantation (B: brain tissue; T: tumour tissue). Scale bar = 2 mm (**c**,**d**); 1 mm (**e**,**f**); 100 μm ((**f**) I–VI).

A total of 1 × 10^6^ C57BL/6 (B6) ESCs, iPSCs, NSCs, iNSCs or MSCs were separately transplanted into the motor cortex of syngeneic adult mouse with normal immunologic function. We chose the motor cortex as target site because tumour formation-induced behavioural changes could be observed at an early stage post-implantation. During the 28-day observation period, mice survived well without any death after NSC, iNSC and MSC transplantation (Fig. 1a). However, ESC and iPSC grafts clearly decreased mouse survival and resulted in massive animal death within 28 days. For example, more than 50% and 90% of mice that received ESC transplants died within 14 and 28 days, respectively. Meanwhile, over 40% and 85% of mice that received iPSC implantation died within 14 and 28 days, respectively. There was no obvious difference in the survival rate between the ESC and iPSC groups (median survival 13 days in the ESC group versus 14 days in the iPSC group). In addition, we observed long-term survival in mice receiving NSC, iNSC and MSC transplants (Fig. 1b). During the 24-week observation period, there was no significant difference in the survival rate among the three groups.

Mouse brain tissues showed no distinctive features in the NSC, iNSC, MSC and Control groups at 14 and 28 days after injection (Fig. 1c). However, brain tissues from the majority of mice in the ESC and iPSC groups showed different degrees of macroscopic malformation (Fig. 1c,d). Moreover, brain tumours and tissue destruction were observed by histological examination. In brief, four types of outcomes were mainly observed post-implantation: (A) malignant teratoma formation, which was identified by the presence of immature tissues from the three embryonic germ layers (ectoderm, mesoderm and endoderm), and severe brain injury accompanied by massive immune cell infiltration (Fig. 1e); (B) benign teratoma formation, which was composed of well-differentiated derivations from the three embryonic germ layers, and mild brain injury accompanied by minor immune cell infiltration (Fig. 1f); (C) borderline tumour formation, with characteristics were between those of malignant and benign teratomas (Supplementary Figure S3a,d); and (D) no evidence of tumour formation, brain injury or immune rejection (Supplementary Figure S3e,h). In contrast, serial HE-stained sections covering the entire brain failed to show any tumour formation in the NSC, iNSC and MSC groups.

The pathological features of the brains were assessed by two independent board-certified neuropathologists, who were blinded to the findings, and classified into three grades based on the characteristics of the brain tumours^21^,^22^,^23^,^24^ and the degrees of brain injury and immune cell infiltration (Supplementary Figure S4 and Supplementary Table S1). We calculated the percent of brain tumours within 14 and 28 days after ESC or iPSC transplantation and found that the tumour-positive detection rate was markedly higher than the tumour-negative detection rate. Moreover, the grade 2 tumour ratios of all cases were the highest in the iPSC group, as well as in the ESC group. In addition, our data revealed that tumour weight as well as lesion size in the brain tissues of mice with grade 3 tumours were markedly larger than in those with grade 2 and 1 tumours. Furthermore, at the same grade, the incidence of brain tumours, tumour weight, and lesion size showed no significant difference between the ESC and iPSC groups.

### Immune cell infiltration in the brain after stem cell transplantation

**Figure 2:**
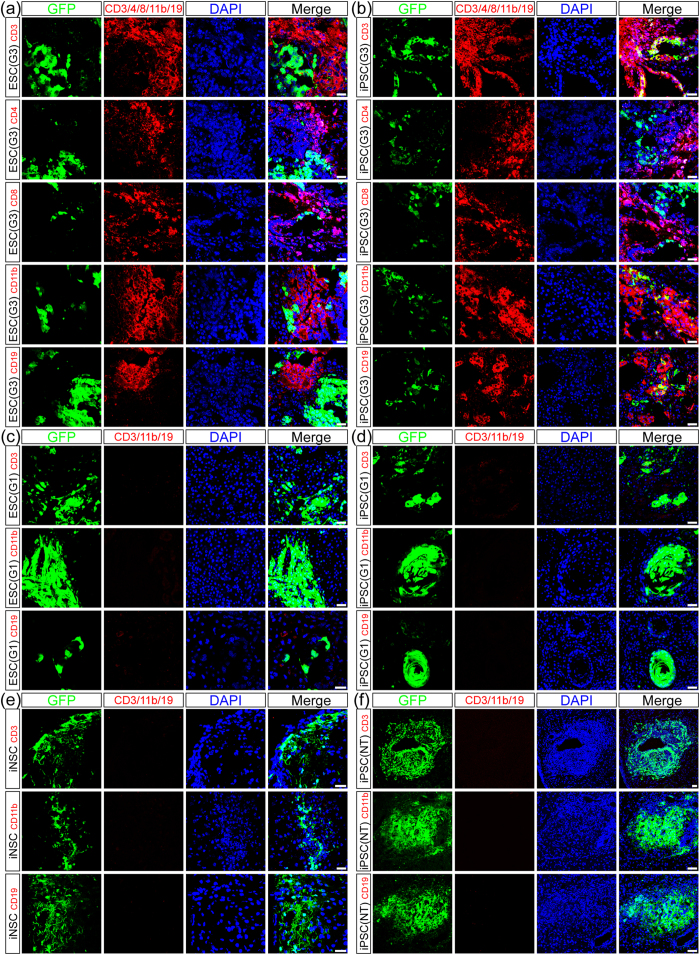
Immune cell infiltration in the brain after stem cell transplantation. (**a**,**b**) Massive numbers of CD3-positive T cells (red), CD4-positive Th cells (red), CD8-positive CTL cells (red), CD11b-positive microglia/macrophages (red) and CD19-positive B cells (red) infiltrated the brains of mice with grade 3 tumours after ESC (green, **a**) or iPSC (green, **b**) transplantation. (**c**,**d**) A few CD3-positive T cells (red), CD11b-positive microglia/macrophages (red) and CD19-positive B cells (red) infiltrated in brains of mice with grade 1 tumours after ESC (green, **c**) or iPSC (green, **d**) transplantation. (**e**,**f**) These immune cells (red) were rarely detected in the brains of mice without tumour formation after iNSC (green, **e**) or iPSC (green, **f**) transplantation. Scale bar = 25 μm.

To clarify the types of infiltrated immune cells, we utilized specific antibodies to detect CD11b-positive microglia/macrophages, CD3-, CD4- and CD8-positive T cells and CD19-positive B cells in the brain within 28 days after stem cell transplantation (Fig. 2 and Supplementary Figure S5). All of these immune cells types were found to infiltrate throughout the brains of mice that developed tumours after ESC or iPSC transplantation. CLSM revealed clear GFP expression in brain tumours derived from ESC and iPSC grafts. Hence, immune cells labelled with specific antibodies could be distinguished from tumour tissues and stem cell grafts. Moreover, the numbers of these immune cells, which were mostly located in the developing tumours and damaged brain regions, were positively correlated with the malignant grades of tumours and the severity of brain injury.

**Figure 3:**
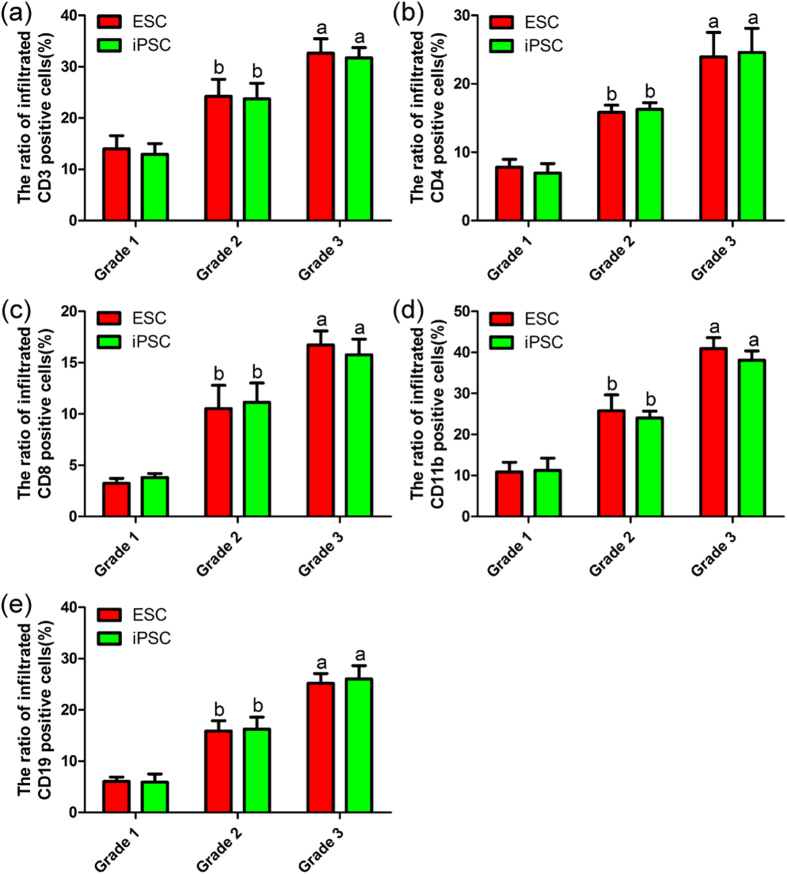
Statistic graphs of the infiltrated immune cells. Data were represented as mean ± SD of six different microscopic fields from three experiments ((**a**) *P* < 0.05 versus grade 2 or grade 1; (**b**) *P* < 0.05 versus grade 1).

For example, mouse brain tissues were riddled with immune cells and accompanied by extensive tissue destruction in the ESC (G3) and iPSC (G3) groups (Fig. 2a,b). However, the numbers of infiltrated immune cells were relatively lower in the brains of mice with grade 2 and 1 tumours after ESC or iPSC transplantation (*P* < 0.05) (Figs 2c,d and 3). Moreover, no significant differences in the numbers of infiltrated immune cells were found between the ESC and iPSC groups at the same tumour grade. In addition, GFP-labelled iNSCs were found to persist in the brain for four weeks without any evidence of tumour formation or immune rejection (Fig. 2e). Furthermore, the aforementioned immune cells were rarely detected in the ESC (NT), iPSC (NT) (Fig. 2f), NSC, MSC and Control groups.

### Immunogenicity of stem cells

**Figure 4:**
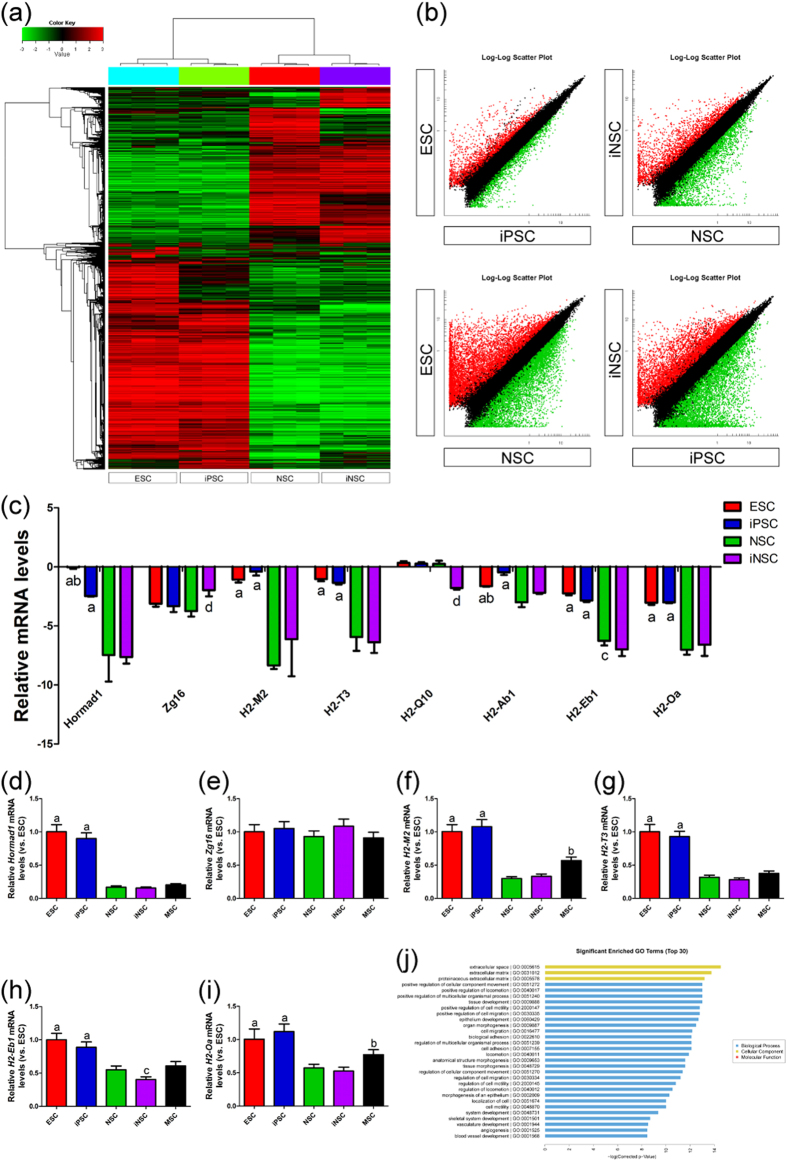
Immunogenicity of stem cells. (**a**,**b**) The global gene expression profiles of ESCs, iPSCs, NSCs and iNSCs were determined using Agilent mRNA expression arrays (n = 3/group). (**c**) The levels of immunogenicity-associated genes, including *Hormad1, Zg16, H2-M2, H2-T3, H2-Q10, H2-Ab1, H2-Eb1* and *H2-Oa* were analyzed after signal values normalization (n = 3/group; (**a**) *P* < 0.05 versus NSCs or iNSCs; (**b**) *P* < 0.05 versus iPSCs; (**c**) *P* < 0.05 versus iNSCs; (**d**) *P* < 0.05 versus ESCs, iPSCs or NSCs). (**d**–**i**) The expression of *Hormad1* (**d**), *Zg16* (**e**), *H2-M2* (f), *H2-T3* (**g**), *H2-Eb1* (**h**) and *H2-Oa* (**i**) genes in ESCs, iPSCs, NSCs, iNSCs and MSCs were determined by qRT-PCR (n = 3/group; (**a**) *P* < 0.05 versus NSCs, iNSCs or MSCs; (**b**) *P* < 0.05 versus NSCs or iNSCs; (**c**) *P* < 0.05 versus NSCs or MSCs). (**j**) Significant enriched GO terms (Top 30) of ESCs, iPSCs, NSCs and iNSCs were determined by GO enrichment analysis (n = 3/group).

To clarify the stimuli that lead to immune cell infiltration, we first examined the immunogenicity of stem cell grafts^7^,^8^. The global gene expression profiles of ESCs, iPSCs, NSCs and iNSCs were determined using Agilent mRNA expression arrays (Fig. 4a,b). IPSCs clustered closely with ESCs, whereas iNSCs clustered closely with NSCs. Most of the genes that were upregulated in ESCs and iPSCs were downregulated in NSCs and iNSCs, and vice versa. Then, we analyzed the normalized signal values of each sample and found that the expression of immunogenicity-associated genes (*Zg16, H2-M2, H2-T3, H2-Q10, H2-Eb1* and *H2-Oa*) was similar between ESCs and iPSCs (Fig. 4c). Moreover, *Hormad1, H2-M2, H2-T3, H2-Ab1, H2-Eb1* and *H2-Oa* expression levels in NSCs and iNSCs were lower than those in ESCs and iPSCs.

To verify the microarray results, we analyzed the expression of *Hormad1, Zg16, H2-M2, H2-T3, H2-Eb1* and *H2-Oa* in ESCs, iPSCs, NSCs, iNSCs and MSCs by qRT-PCR (Fig. 4d,i). The relative levels of *Hormad1, H2-M2, H2-T3, H2-Eb1* and *H2-Oa* genes in NSCs, iNSCs and MSCs were obviously lower than those in ESCs and iPSCs. However, *Zg16* levels had no significant differences in these stem cells. Besides, the relative levels of *H2-M2* and *H2-Oa* genes in MSCs were markedly higher than those in NSCs and iNSCs. Moreover, *H2-Eb1* levels in iNSCs were obviously lower than those in NSCs and MSCs. In short, the qRT-PCR results were basically in agreement with the microarrays and revealed that these immunogenicity-associated genes were weakly expressed in NSCs, iNSCs and MSCs.

**Figure 5:**
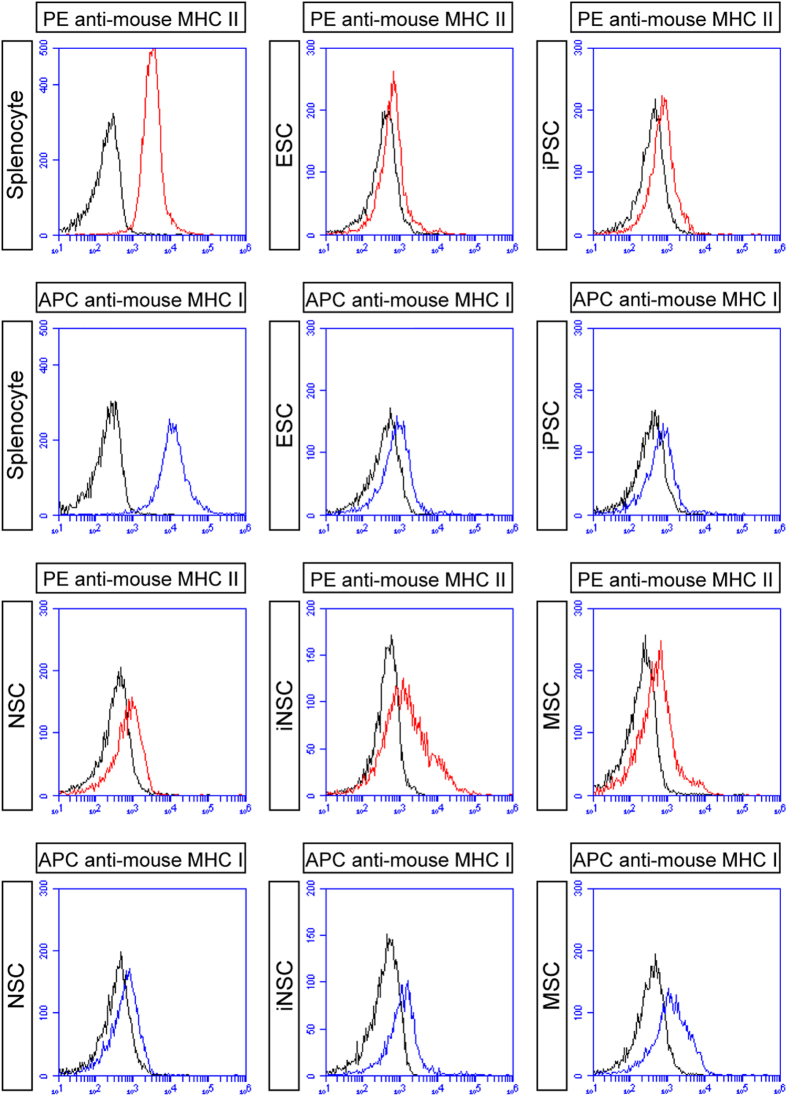
Flow cytometry analyses. The levels of MHC-class I (blue) and II (red) showed no obvious intergroup differences among ESCs, iPSCs, NSCs, iNSCs and MSCs. Moreover, the levels of MHC-class I and II in these stem cells were lower than those in syngeneic splenocytes (n = 3/group). Isotype antibodies were used as controls (black).

To further explore the immunogenicity of ESCs, iPSCs, NSCs, iNSCs and MSCs, we detected MHC-class I and II molecule expression levels in these stem cells by flow cytometry and used syngeneic splenocytes as positive controls (Fig. 5). Flow cytometry analyses revealed that MHC-class I and II expression showed no significant intergroup differences among these stem cells. Moreover, the levels of MHC-class I and II in these stem cells were lower than those in syngeneic splenocytes.

### The expression of chemokines CCL5 and CXCL12

**Figure 6:**
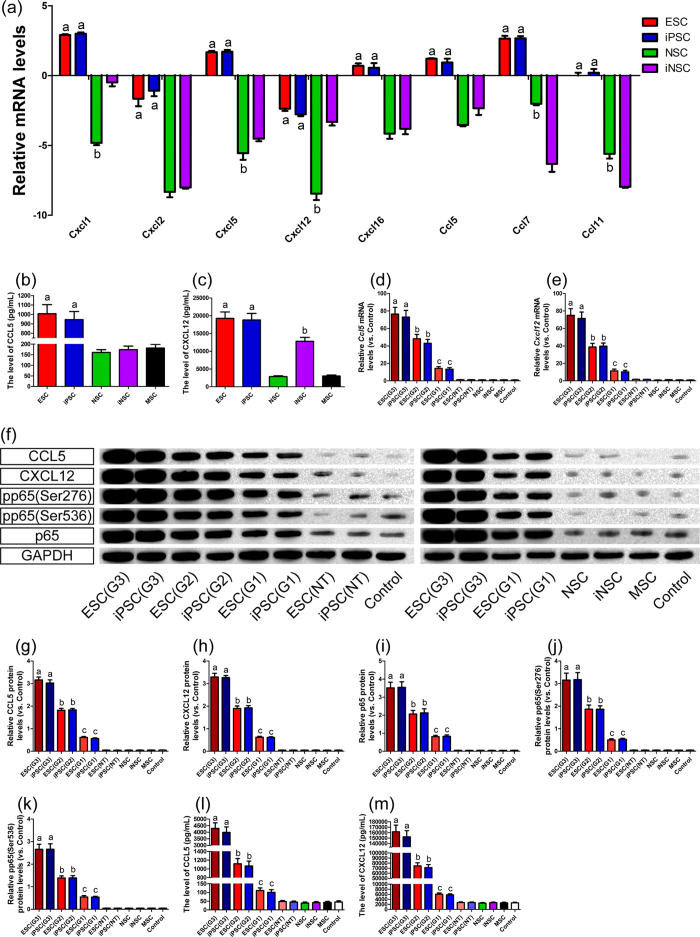
The expression of chemokines and the activation of NF-κB. (**a**) The expression of chemokine-related genes, including *Ccl5/7/11* and *Cxcl1/2/5/12/16*, was analyzed after signal values normalization (n = 3/group; ((**a**) *P* < 0.05 versus NSCs or iNSCs; (**b**) *P* < 0.05 versus iNSCs). (**b**,**c**) CCL5 and CXCL12 levels in the culture supernatants of these stem cells were detected by ELISA (n = 6/group; (**a**) *P* < 0.05 versus NSC, iNSC or MSC; (**b**) *P* < 0.05 versus NSC or MSC). (**d**,**e**) *Ccl5* and *Cxcl12* gene expression in brain tissues after stem cell transplantation was analyzed by qRT-PCR (n = 3/group; ((**a**) *P* < 0.05 versus ESC (G2), iPSC (G2), ESC (G1), iPSC (G1), ESC (NT), iPSC (NT), NSC, iNSC, MSC or Control; (**b**) *P* < 0.05 versus ESC (G1), iPSC (G1), ESC (NT), iPSC (NT), NSC, iNSC, MSC or Control; (**c**) *P* < 0.05 versus ESC (NT), iPSC (NT), NSC, iNSC, MSC or Control). (**f**) Representative immunoblots depicted the levels of CCL5, CXCL12, p65, p65 phosphorylated at Ser276 and Ser536 in the brain. (**g**-**m**) Histograms showed that the levels of CCL5 (**g**), CXCL12 (**h**), p65 (**i**), p65 phosphorylated at Ser276 (**j**) and Ser536 (**k**) in the brain, serum CCL5 (l) and CXCL12 (**m**) (n = 3/group (WB), n = 6/group (ELISA); ((**a**) *P* < 0.05 versus ESC (G2), iPSC (G2), ESC (G1), iPSC (G1), ESC (NT), iPSC (NT), NSC, iNSC, MSC or Control; (**b**) *P* < 0.05 versus ESC (G1), iPSC (G1), ESC (NT), iPSC (NT), NSC, iNSC, MSC or Control; (**c)** *P* < 0.05 versus ESC (NT), iPSC (NT), NSC, iNSC, MSC or Control).

Given the lack or low expression of immunogenicity-associated genes in these stem cell grafts, the infiltration of immune cells in syngeneic mouse brains could be caused by other stimuli. Based on the global gene expression profiles of these stem cells, we then performed gene ontology (GO) enrichment analysis to map obviously enriched GO terms (Fig. 4j). Among the top fits, we found 16 chemokine-related genes that were over-expressed in ESCs and iPSCs but underexpressed in NSCs and iNSCs (Fig. 6a).

Thus, we speculated that immune cell recruitment might be associated with the role of chemokines. We conducted ELISA to detect the levels of CCL5 and CXCL12 in the culture supernatants of these stem cells (Fig. 6b,c). Higher levels of CCL5 expression were found in ESCs and iPSCs, along with lower expression levels in NSCs, iNSCs and MSCs. Furthermore, there was no significant difference in the secretion of CCL5 between ESCs and iPSCs. CXCL12 levels were also similar between ESCs and iPSCs and were markedly higher in ESCs and iPSCs than in NSCs, iNSCs and MSCs. Moreover, iNSCs clearly released more CXCL12 than either NSCs or MSCs did (*P* < 0.05).

Next, we performed qRT-PCR to analyze the expression of the *Ccl5* and *Cxcl12* in brain tissues after stem cell transplantation (Fig. 6d,e). *Ccl5* and *Cxcl12* were strongly expressed in the brains of mice that developed tumours after ESC or iPSC transplantation; the expression levels of these genes were also positively correlated with the malignant grades of the tumours. *Ccl5* gene expression was increased 217-fold in the ESC group (G3) and 208-fold in the iPSC group (G3) compared with the control. Grade 3 tumours expressed *Ccl5* at levels that were 3-, 31- and 78-fold higher compared with grade 2, 1 tumours and no tumour, respectively, in the ESC and iPSC groups. *Ccl12* gene expression was increased 152-fold in the ESC (G3) and 143-fold in the iPSC (G3) compared with the control. Grade 3 tumours expressed *Ccl12* gene at levels that were 2-, 22- and 62-fold higher compared with grade 2, 1 tumours and no tumour, respectively, in the ESC and iPSC groups.

In addition, western blotting revealed that CCL5 and CXCL12 protein levels were significantly higher in the brain tissues of mice that developed tumours after ESC or iPSC transplantation (*P* < 0.05, Fig. 6f,h). Furthermore, CCL5 and CXCL12 levels in the brain tissues of mice with grade 3 tumours were higher than in those with grade 2 and 1 tumours. At the same tumour grade, there were no obvious differences in CCL5 and CXCL12 levels between the ESC and iPSC groups. Moreover, CCL5 and CXCL12 levels were very low in the ESC (NT), iPSC (NT), NSC, iNSC, MSC and Control groups.

### The activation of NF-κB

To clarify the mechanism of elevated CCL5 and CXCL12 levels in brain tumour tissues, we observed the activation of NF-κB in these tissues using western blot analysis (Fig. 6I,k). The expressions of p65, p65 phosphorylated at Ser276 and Ser536 were markedly higher in the brain tissues of mice that developed tumours than in those without tumour formation and were positively correlated with the malignant grades of the tumours (*P* < 0.05). Moreover, at the same tumour grade, there were no significant differences between the ESC and iPSC groups in the levels of p65 and phosphorylated p65. Furthermore, the levels of p65 and phosphorylated p65 were similarly low among the ESC (NT), iPSC (NT), NSC, iNSC, MSC and Control groups.

### The levels of serum CCL5 and CXCL12

Given that the infiltration of immune cells driven by CCL5 and CXCL12 chemotaxis may be affected by blood-brain barrier (BBB), we detected the serum concentrations of CCL5 and CXCL12 (Fig. 6l,m). Serum CCL5 and CXCL12 levels in mice with brain tumours were obviously higher than those in mice without tumour formation and were positively correlated with the malignant grades of the tumours (*P* < 0.05). Moreover, at the same tumour grade, there was no significant difference in the serum levels of CCL5 and CXCL12 between ESC and iPSC transplantation. In addition, the serum concentrations of CCL5 and CXCL12 were similarly low among the ESC (NT), iPSC (NT), NSC, iNSC, MSC and Control groups.

## Discussion

Currently, stem cell transplantation holds great promise for the treatment of central nervous system (CNS) diseases; thus, the safety of these grafts in the CNS should be a primary concern^3^,^4^. In this study, we mainly determined the tumourigenicity and immunogenicity of ESC, iPSC, NSC, iNSC and MSC grafts in syngeneic mouse brains. We demonstrate that iPSCs, as well as ESCs, can form tumours in mouse brains, leading to tissue destruction and immune cell infiltration. In contrast, we report no evidence of tumour formation, brain injury or immune rejection in the iNSC, NSC, and MSC groups. Therefore, iNSC grafts are safer than iPSC grafts, with no resulting tumourigenicity or immunogenicity.

As mentioned above, we chose syngeneic mouse brain receiving stem cell grafts as a studied system for several reasons. First, our purpose was to evaluate the safety of stem cell grafts in the CNS. Besides, studying the tumourigenicity and immunogenicity of various stem cell grafts in the specific brain microenvironment has great significance^3^,^25^. However, few studies on these issues have been performed comparing iNSCs and iPSCs. In addition, because of the limited intracranial space, developing brain tumours can lead to increased intracranial pressure and tissue destruction, which are associated with a high mortality rate^24^,^26^. Therefore, we were able to identify tumour formation by observing behavioural changes in the mice (data not shown). Moreover, immune responses in the CNS also have many characteristics, and their mechanisms are complicated by the influence of BBB, which is also currently an area of intense research^27^,^28^. Thus, the brains of syngeneic mice with normal immunologic function are suitable for this study.

To compare the tumourigenicity of stem cell grafts, we first classified and determined the percentage of brain tumours at different grades. This novel method provides a platform for a more systematic and comprehensive analysis of the tumourigenicity of these grafts than ever before^7^. It suggests that the detection rate of brain tumours is not 100% even though ESC and iPSC grafts retain the potential for tumour formation. In addition, the same stem cells can form a variety of tumour types, including benign, borderline and malignant tumours, in the brain. Our results are partially consistent with previous reports^7^,^8^,^9^, and the discrepancies may be due to the complicated effects of brain microenvironment on the behaviour of stem cell grafts^4^,^27^. Based on analyses of global gene expression profiles, pluripotency is likely major reason for the apparent differences between iPSCs and iNSCs in terms of tumour formation. Therefore, reprogramming technology does not increase the propensity for tumour formation in iNSC-based therapy, which is consistent with previous studies (Supplementary Figure S6)^14^,^15^,^18^.

We next determined whether these stem cell grafts could cause immunologic rejection in syngeneic mouse brains. Our findings differ from those of previous reports^7^,^8^,^9^, which can be summarized and analyzed as follows. Initially, immune cell recruitment is still visible in the brains of mice that develop tumours after receiving syngeneic ESC grafts, which should evade immune rejection. In addition, the numbers of infiltrated immune cells at the same tumour grade does not differ markedly between the ESC and iPSC groups. What is more, as mentioned above, elevated numbers of immune cells positively correlated with high-grade tumours and severe brain injury. Moreover, analyses of global gene expression profiles, qRT-PCR and flow cytometry suggest that immunogenicity-associated genes (*Hormad1, Zg16, MHC-class I* and *MHC-class II*) are weakly expressed in these stem cells and at similar levels in ESCs and iPSCs. Here, we firstly assume that the discrepancies between our results and those of previous studies might be due to genetic or epigenetic instability caused by reprogramming technology^20^. If this explanation is true, reprogramming technology does not cause every cell to stably express these genes (*Hormad1* and *Zg16*). Conversely, if this assumption is invalid, then reprogramming technology does not lead to genetic or epigenetic instability, and these genes are not abnormally expressed in stem cells. Thus, immune cell infiltration may not be due to the immunogenicity of these stem cell grafts but rather to tissue destruction, which is caused by the development of ESC- or iPSC-derived brain tumours.

Tumours not only induce tissue damage, but also release a variety of substances to recruit immune cells. Here, we mainly focused on chemokines, which are major regulators of cell proliferation, activation, trafficking and adhesion^29^,^30^. Among these chemokines, CCL5 and CXCL12, which are associated with tissue destruction, are involved not only in tumour progression, angiogenesis and metastasis, but also in immune cell infiltration^30^,^31^,^32^. Hence, CCL5 and CXCL12 regulate the migration of immune cells into the developing tumour and trauma region^30^,^31^,^32^,^33^. We found that in addition to the chemokines secreted by engrafted stem cells, brain tumours derived from these grafts also produced high levels of CCL5 and CXCL12.

The transcription of CCL5 and CXCL12 are reported to be largely modulated via NF-κB signalling, which plays important roles in the inhibition of apoptosis in many tumour types^30^,^31^,^32^. Meanwhile, CCL5 and CXCL12 can also regulate the activation of NF-κB^32^. Based on our results, the interactions between chemokines and NF-κB signalling may constitute a system that leads to the constantly rising levels of CCL5, CXCL12, p65 and phosphorylated p65. This system has multiple roles in the inhibition of apoptosis in tumours, but at the same time may induce immune cell recruitment^30^,^31^,^32^,^33^. In the present study, we found that immune cells broke through the dysfunctional BBB after brain injury. As we know, these immune cells have the potential to secrete pro-inflammatory cytokines to enhance NF-κB activation^33^,^34^. Thus, CCL5 and CXCL12 levels in brain tissue and serum will continue to rise rapidly.

## Methods

### Cell

#### B6 MEFs

MEFs were isolated from the bodies of embryonic day 13.5 B6 mice (Vital River Laboratories, Beijing, China) by several cycles of 0.25% trypsin-EDTA (Invitrogen, Carlsbad, CA, USA) digestion and plated at a density of 1 × 10^6^ cells/ml in MEF culture medium (MEFcm, DMEM containing 10% fetal bovine serum (FBS) and 0.1 mM non-essential amino acids (NEAA)) (all from Invitrogen). After reaching 90% confluence, cells were inactivated by Mitomycin C (MMC, Sigma-Aldrich, St. Louis, MO, USA) as described^18^ and used as feeders to culture ESCs or iPSCs.

#### B6 GFP/rtTA ESCs and iPSCs

B6 GFP/rtTA ESCs and iPSCs (Oct4/Sox2/Klf4/c-Myc) were generated as described previously and plated on MMC-treated B6 MEF feeders in ESC/iPSC culture medium (ESC/iPSCcm, DMEM containing 15% FBS, 0.1 mM NEAA, 1% Nucleosides (Invitrogen), 2 mM L-glutamine, 10 ng/ml leukemia inhibitory factor (LIF, Invitrogen) and 0.1 mM β-Mercaptoethanol (Sigma-Aldrich))^18^. Cells were dissociated using accutase (Invitrogen) and plated on 0.1% gelatin-coated dishes for 0.5 h to remove feeders prior to harvesting.

#### B6 NSCs

NSCs were isolated from the forebrain region of embryonic day 13.5 B6 mice by several cycles of 0.05% trypsin-EDTA digestion and plated at a density of 5 × 10^5^ cells/mL in NSC culture medium (NSCcm, Neurobasal: DMEM/F12 (1:1) containing 2% B27 supplements, 20 ng/ml basic fibroblast growth factor (bFGF) and 20 ng/ml epidermal growth factor (EGF)) (all from Invitrogen).

#### B6 GFP/rtTA iNSCs

B6 GFP/rtTA iNSCs, directly generated by transcription factor-mediated reprogramming (Oct4/Sox2/Klf4/c-Myc) without the derivation of iPSCs, were generated as described previously and plated at a density of 5 × 10^5^ cells/mL in iNSC culture medium (iNSCcm, Neurobasal: DMEM/F12 (1:1) containing 2% B27 supplements, 20 ng/ml bFGF, 20 ng/ml EGF, 0.05% bovine serum albumin (BSA) and 2 mM L-glutamine) (all from Invitrogen)^18^.

#### B6 MSCs

MSCs were isolated from the femora and tibia of adult B6 mouse and harvested by Percoll (Sigma-Aldrich) gradient centrifugation at a density of 1.082 g/ml. Cells were plated at a density of 5 × 10^5^ cells/mL in MSC culture medium (MSCcm, DMEM/F12 (1:1) containing 10% FBS and 2 mM L-glutamine).

### Identification of cultured cells

For identification, cells were dissociated using accutase and seeded on glass coverslips coated with poly-l-lysine (PLL) (Sigma-Aldrich) on a 24-well plate (1 × 10^5^ cells per well) in culture medium for several days. Then cells were fixed with 4% paraformaldehyde (PFA) in 0.1 M PBS (pH 7.4) for 20 min and identified by immunofluorescence staining. Furthermore, MSCs were dissociated and identified by flow cytometry using the characteristics of surface markers CD29, CD44, CD71, CD14, CD34 and CD45.

### Animal

Healthy adult male B6 mice weighing 20~28 g (Vital River Laboratories, Beijing, China) were housed in a temperature- and humidity-controlled room with food and water *ad libitum*. All experimental procedures were in compliance with the Guide for the Care and Use of Laboratory Animals published by the National Institutes of Health (NIH) and approved by the Committee on the Ethics of Animal Experiments of the General Hospital of Beijing Military Region, P.L.A (Permit Number: 2014–044). Mice were randomly assigned to six groups: the NSC group (mice receiving NSC transplantation), the iNSC group (mice receiving iNSC transplantation), the ESC group (mice receiving ESC transplantation), the iPSC group (mice receiving iPSC transplantation), the MSC group (mice receiving MSC transplantation) and the Control group (mice receiving PBS treatment).

For transplantation, cultured cells were digested with accutase and washed three times with PBS. After the density of single-cell suspension was adjusted, the cells were maintained on ice. Animals were anaesthetized with thiopental (40 mg/kg body weight, intraperitoneal injection) and then mounted in a stereotaxic apparatus (Stoelting, Wood Dale, IL, USA). After the mouse skin was shaved and cleaned, a midline scalp incision was made to expose the mouse parietal bones. Cell suspension or PBS was separately injected into the brain (5.5 mm anterior to the lambda suture, 1.0 mm lateral to the middle line and 2.0 mm under the dura) using different sterile 25-μl 22 S Hamilton syringes. Each site received 5 μl of cell suspension containing 1 × 10^6^ cells or PBS at a speed of 0.5 μl/min. Approximately 5 min after injection, the syringe was slowly withdrawn. Animals received fentanyl (0.05 mg/kg body weight per day, intraperitoneal injection) as the analgesic agent post operation. On days 14 and 28 after implantation, venous blood was drawn from the right atrium of mice under anaesthesia. Animals were sacrificed with thiopental overdose, and their fresh or perfused-fixed brain and spleen tissues were collected for morphological and molecular-biological analyses.

### Morphological analysis

Brain and spleen tissues were postfixed in 4% PFA in 0.1 M PBS (PH 7.4) at 4 °C overnight, and then sectioned (10 μm) on a cryostat (Leica CM 1950, Leica Biosystems, Nussloch, Germany) and mounted on adhesion microscope slides. The pathologic features of brain tissues were assessed by hematoxylin and eosin (HE) staining.

### Immunofluorescence

Slides of brain and spleen tissues, as well as of cultured cells, were blocked for 1 h using 10% BSA/0.3% TritonX-100 and then incubated overnight at 4 °C with primary antibodies (Supplementary Table S2). After being washed in PBS, they were incubated for 1~2 h at room temperature (RT) with secondary antibodies (Supplementary Table S2). After several washes with PBS, the nuclei were stained with Dapi Fluoromount-G (SouthernBiotech, Birmingham, AL, USA) and staining was detected via fluorescent microscopy (DM3000, Leica) or confocal laser scanning microscopy (CLSM, TCS SP5 II, Leica). The number of positive cells was manually counted directly on the microscopy at 20 x magnification and adjusted using image analysis software (Image-Pro plus 5.0). The ratio of positive cells was calculated as (number of positive cells/total number of cells) × 100%.

### TUNEL staining

TUNEL staining was performed using the *In Situ* Cell Death Detection Kit, TMR red (Roche, Mannheim, Germany) as described in the manufacturer’s instructions. The nuclei were counterstained with Dapi Fluoromount-G and staining was detected via fluorescent microscopy. The ratio of TUNEL-positive cells was calculated as (number of TUNEL-stained cells/total number of DAPI-stained cells) × 100%.

### Agilent mRNA expression array and qRT-PCR assay

The Agilent mRNA expression array was performed by the CapitalBio Company (Beijing, China). For verification, RNA extracted from cultured cells and brain tissues was reversely transcribed into cDNA using the QuantScript RT kit (Tiangen Biotech, Beijing, China) and assessed by qRT-PCR assay using the SYBR-Green Master Mix (TaKaRa Biotech, Dalian, China) and a ViiA7 Real-Time PCR System (Applied Biosystems, Foster City, CA, USA). The sequences of the PCR primer pairs used in this study were reported previously^7^,^18^,^35^.

### ELISA

Cell culture supernatants were purified by centrifugation for 20 min at 3000 r.p.m and stored at −80 °C. Serum was separated from the venous blood using the BD Vacutainer SST^TM^ tubes (BD Biosciences, San Jose, CA, USA) after centrifugation for 20 min at 3000 r.p.m and stored at −80 °C. CCL5 and CXCL12 levels were detected in duplicate assays using ELISA kits (R&D Systems, Minneapolis, MN, USA) according to the manufacturer’s protocols.

### Western blot

Protein was extracted from brain tissues using the RIPA reagent (Sigma-Aldrich) supplemented with protease and phosphatase inhibitors (Fermentas, Burlington, Canada). Protein concentrations were determined using the BCA assay (Thermo Scientific, Hudson, NH, USA). Protein samples were heated for 10 min at 95 °C and separated by SDS-PAGE (35 μg per lane), and then transferred to PVDF membranes (Millipore, Bedford, MA, USA). The blots were blocked for 1 h at RT with 5% BSA in TBST and then detected by incubation with primary antibodies (Supplementary Table S2) at 4 °C overnight. After several washes, the blots were incubated for 1 h at RT with HRP-conjugated secondary antibodies (Supplementary Table S2). Immunoblots were visualized using the SuperSignal ECL (Pierce, Rockford, IL, USA). The results expressed relative to the control and normalized to GAPDH.

### Flow cytometry

After digestion, the Fc receptors of cultured cells were blocked with TruStain fcXTM reagent (Biolegend, San Diego, CA, USA) for 10 min on ice. Then cells were incubated with cell surface antibodies (Supplementary Table S2) for 30 min at 4 °C. After several washes, cells were resuspended in PBS and analyzed on an Accuri C6 Flow Cytometer System (BD Biosciences). Fluorescent isotype antibodies (Supplementary Table S2) were used at the same concentrations as controls.

### Statistical analysis

The SPSS17.0 statistical software package was used for statistical analysis. Data were presented as mean ± standard deviation (SD). Kaplan-Meier survival analysis, student’s t-test, one-way ANOVA, Chi-Square test and rank sum test were used to determine statistical significance. A *P* < 0.05 was considered statistically significant.

## Additional Information

**How to cite this article**: Gao, M. *et al*. Tumourigenicity and Immunogenicity of Induced Neural Stem Cell Grafts Versus Induced Pluripotent Stem Cell Grafts in Syngeneic Mouse Brain. *Sci. Rep.* **6**, 29955; doi: 10.1038/srep29955 (2016).

## Supplementary Material

Supplementary Information
