# Supplementary material for: Tumourigenicity and Immunogenicity of Induced Neural Stem Cell Grafts Versus Induced Pluripotent Stem Cell Grafts in Syngeneic Mouse Brain
Source: Sci Rep. 2016 Jul 15;6:29955. doi: 10.1038/srep29955 (PMC4945932; doi:10.1038/srep29955)
Supplement: Supplementary Information [file srep29955-s1.pdf]

Supplementary material for: Tumourigenicity and Immunogenicity of Induced Neural Stem Cell Grafts Versus Induced Pluripotent Stem Cell Grafts in Syngeneic Mouse Brain

Mou Gao [a, b], Hui Yao [a,*], Qin Dong [c], Hongtian Zhang [a], Zhijun Yang [a], Yang Yang [a], Jianwei Zhu [a], Minhui Xu [b,*], Ruxiang Xu [a,*]

[a]*Department of Neurosurgery, General Hospital of Beijing Military Region, Beijing 100700, China*

[b]*Department of Neurosurgery, Daping Hospital, Third Military Medical University, Chongqing 400042, China*

[c]*Department of Neurology, Fu Xing Hospital, Capital Medical University, Beijing 100038, China*

Supplementary Table S1: Percent of tumour grades in group ESC and group iPSC

Grade	Standard	ESC group (%)		IPSC group (%)	
		Day 14	Day 28	Day 14	Day 28
NT	1.No tumour formation<br>2.Rare brain injury<br>3.Few immune cell infiltration	2/30 (6.7)	4/42 (9.5)	2/28 (7.1)	5/42 (11.9)
Grade 1 (G1)	1.Benign teratoma formation<br>2.Mild brain injury<br>3.A few immune cell infiltration	8/30 (26.7)	12/42 (28.6)	8/28 (28.6)	13/42 (31.0)
Grade 2 (G2)	1.Borderline tumour formation<br>2.Moderate brain injury<br>3.Localized immune cell infiltration	11/30 (36.7)	14/42 (33.3)	9/28 (32.1)	13/42 (31.0)
Grade 3 (G3)	1.Malignant teratoma formation<br>2.Severe brain injury<br>3.Massive immune cell infiltration	9/30 (30.0)	12/42 (28.6)	9/28 (32.1)	11/42 (26.2)

Supplementary Table S2: Antibodies were used in this study

Specificity	Host	Detection	Dilution	Application	Origin
Nanog	Rabbit	ESC/iPSC	1 μg/ml	IF	Abcam
Nestin	Mouse	NSC/iNSC	4 μg/ml	IF	Millipore
Sox2	Goat	NSC/iNSC	0.5 μg/ml	IF	Santa Cruz
CD44	Rat	MSC	2 μg/ml	IF	Abcam
CD34	Rabbit	MSC	5 μg/ml	IF	Abcam
CD3	Rabbit	T cell	1 μg/ml	IF	Abcam
CD4	Rat	Th cell	0.4 μg/ml	IF	Santa Cruz
CD8	Rat	CTL cell	2 μg/ml	IF	Santa Cruz
CD11b	Rat	Microglia/Macrophage	5 μg/ml	IF	Abcam
CD19	Rat	B cell	2.5 μg/ml	IF	Lifespan
Ki67	Rabbit	iNSC	2 μg/ml	IF	Abcam
NeuN	Mouse	Neuron	5 μg/ml	IF	Millipore
Map2	Rabbit	Neuron	2 μg/ml	IF	Abcam
Mouse IgG	Donkey	Nestin	2 μg/ml	IF	Life Tech
Rat IgG	Donkey	CD44/CD4/CD8/CD11b/CD19	2 μg/ml	IF	Life Tech
Rabbit IgG	Donkey	Nanog/CD34/CD3	2 μg/ml	IF	Life Tech
Goat IgG	Donkey	Sox2	2 μg/ml	IF	Life Tech
Active Caspase-3	Rabbit	Brain	1 μg/ml	WB	Abcam
Bax	Rabbit	Brian	1:1000	WB	CST

CCL5	Rabbit	Brain	1.5 μg/ml	WB	Abcam
CXCL12	Rabbit	Brian	1:1000	WB	Millipore
NF-κB p65	Rabbit	Brian	0.5 μg/ml	WB	Abcam
NF-κB p65 (phosphorS276)	Rabbit	Brian	1 μg/ml	WB	Abcam
NF-κB p65 (phosphoS536)	Rabbit	Brian	0.2 μg/ml	WB	Abcam
GAPDH	Rabbit	Brain	0.2 μg/ml	WB	Santa Cruz
Rabbit IgG	Donkey	CCL5/CXCL12/NF-κB p65/p65(phosphorS276) /pp65(phosphorS536)/GAPDH	0.08 μg/ml	WB	ZSGB-BIO
PE Anti-Mouse CD29		CD29	2 μg/ml	Flow Cyt	eBioscience
PE Armenian Hamster IgG Isotype Control			2 μg/ml	Flow Cyt	eBioscience
FITC Anti-Mouse CD44		CD44	5 μg/ml	Flow Cyt	BD Biosciences
FITC Rat IgG2b, κ Isotype Control			5 μg/ml	Flow Cyt	BD Biosciences
FITC Anti-Mouse CD71		CD71	5 μg/ml	Flow Cyt	BD Biosciences
FITC Rat IgG1, κ Isotype Control			5 μg/ml	Flow Cyt	BD Biosciences
PE Anti-Mouse CD14		CD14	1 μg/ml	Flow Cyt	BD Biosciences
PE Rat IgG1, κ Isotype Control			1 μg/ml	Flow Cyt	BD Biosciences
APC Anti-Mouse CD34		CD34	2 μg/ml	Flow Cyt	BD Biosciences
PE Rat IgG2a, κ Isotype Control			2 μg/ml	Flow Cyt	BD Biosciences
APC Anti-Mouse CD45		CD45	2 μg/ml	Flow Cyt	eBioscience
APC Rat IgG2b, κ Isotype Control			2 μg/ml	Flow Cyt	eBioscience
APC Anti-Mouse H-$2K^b$		MHC-class I molecule	5 μg/ml	Flow Cyt	Biolegend
APC Mouse IgG2a, κ Isotype Control			5 μg/ml	Flow Cyt	Biolegend
PE Anti-Mouse I-A^b		MHC-class II molecule	5 μg/ml	Flow Cyt	Biolegend
PE Mouse IgG2a, κ Isotype Control			5 μg/ml	Flow Cyt	Biolegend

IF: Immunofluorescence; WB: Western blot; Flow Cyt: Flow cytometry

Abcam, Cambridge, MA, USA; Millipore, Bedford, MA, USA; Santa Cruz, Santa Cruz, CA, USA; Lifespan, Seattle, WA, USA; Life Tech, Gaithersburg, MD, USA; CST, Beverly, MA,

USA; ZSGB-BIO, Beijing, China; eBioscience, San Diego, CA, USA; BD Biosciences, San Jose, CA, USA; Biolegend, San Diego, CA, USA

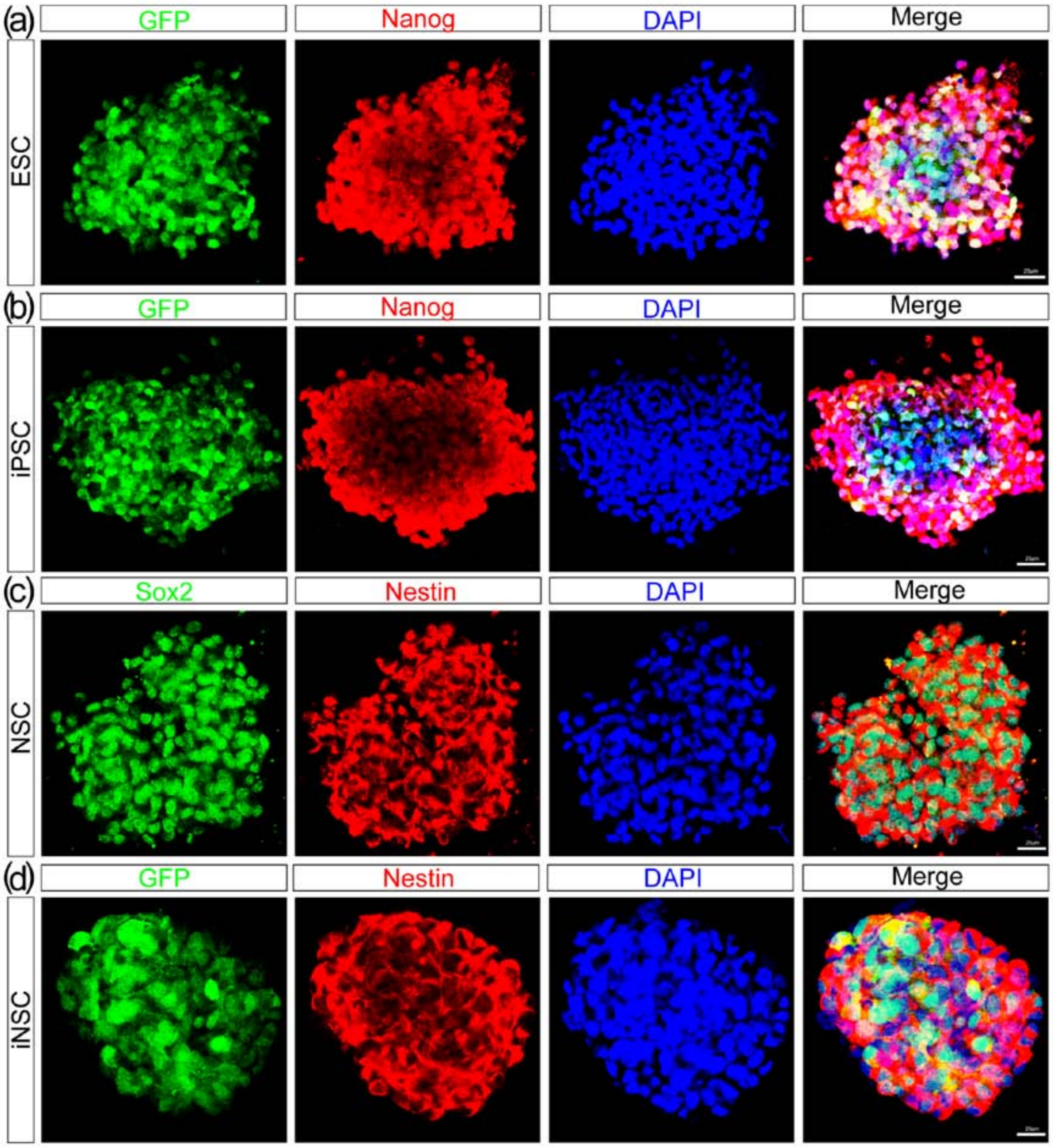

Supplementary Figure S1: Identification and characterization of ESCs, iPSCs, NSCs and iNSCs by immunofluorescence. (A-B) GFP/rtTA ESCs and iPSCs were positive for Nanog (red), merged with nuclei (blue) and GFP (green) via confocal laser scanning microscopy (CLSM). (C) NSCs were positive for Nestin (red) and Sox2 (green), merged with nuclei (blue). (D) GFP/rtTA iNSCs were positive for Nestin (red), merged with nuclei (blue) and GFP (green). Scale bar=25 μm.

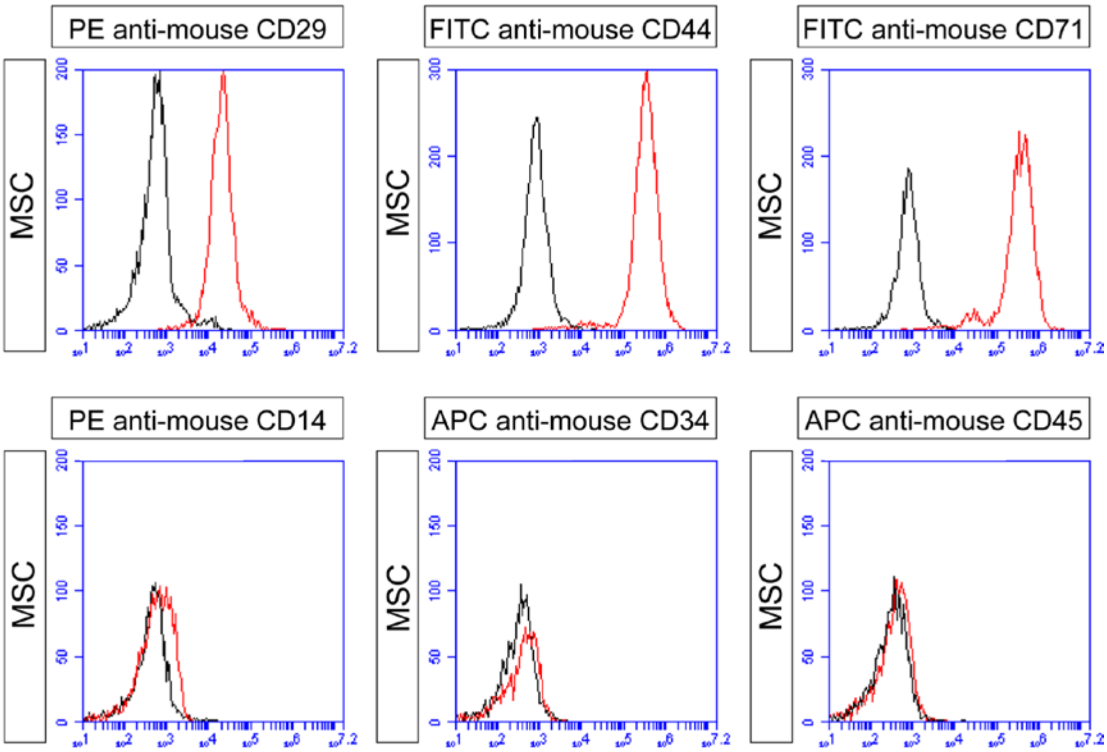

SupplementaryFigure S2: Flow cytometry analyses. MSCs had positive expression of the cell surface markers CD29, CD44 and CD71 (>80%) and low expression of the haematopoietic markers CD14, CD34 and CD45 (<5%).

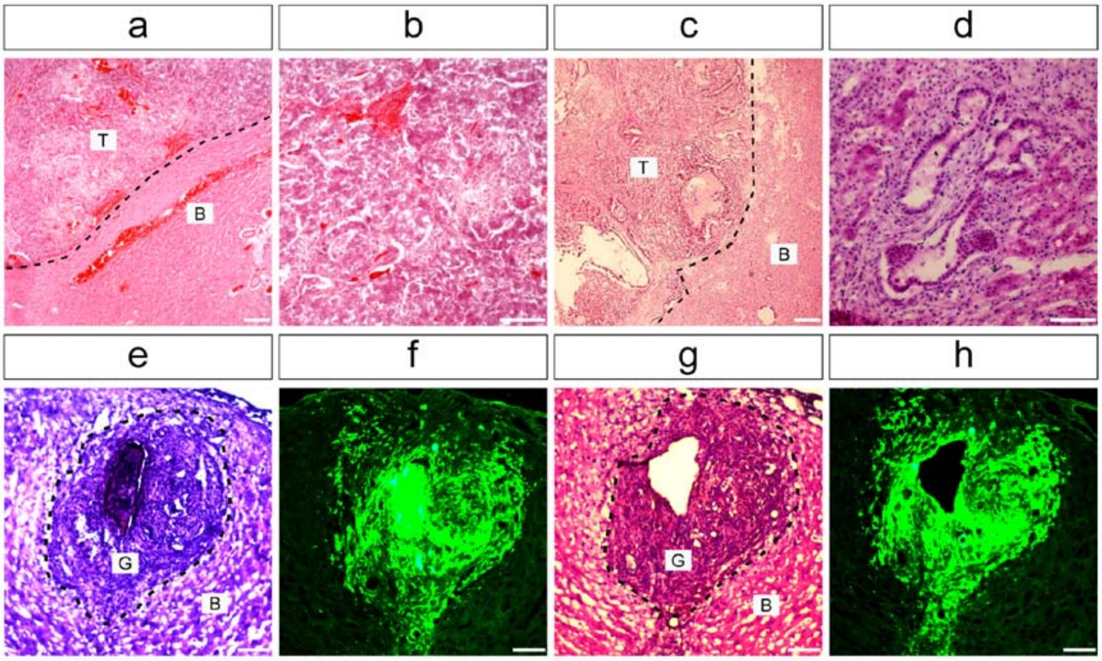

Supplementary Figure S3: Morphology analysis. (a-d) Borderline tumour formation, with characteristics between those of malignant and benign teratomas, was observed in the brain of mice subjected to syngeneic ESC grafts (a: 5 x magnification; b: 20 x magnification) or iPSC grafts (c: 5 x magnification; d: 20 x magnification) on day 14 post-implantation (B: brain tissue; T: tumour tissue). (e-h) No evidence of tumour formation was detected via fluorescence microscopy in bright field images (e, g) or dark field images (f, h) in the brain of mice subjected to syngeneic ESC grafts (e, f) or iPSC grafts (g, h) on day 28 post-implantation (B: brain tissue; G: grafts). Scale bar=50 μm.

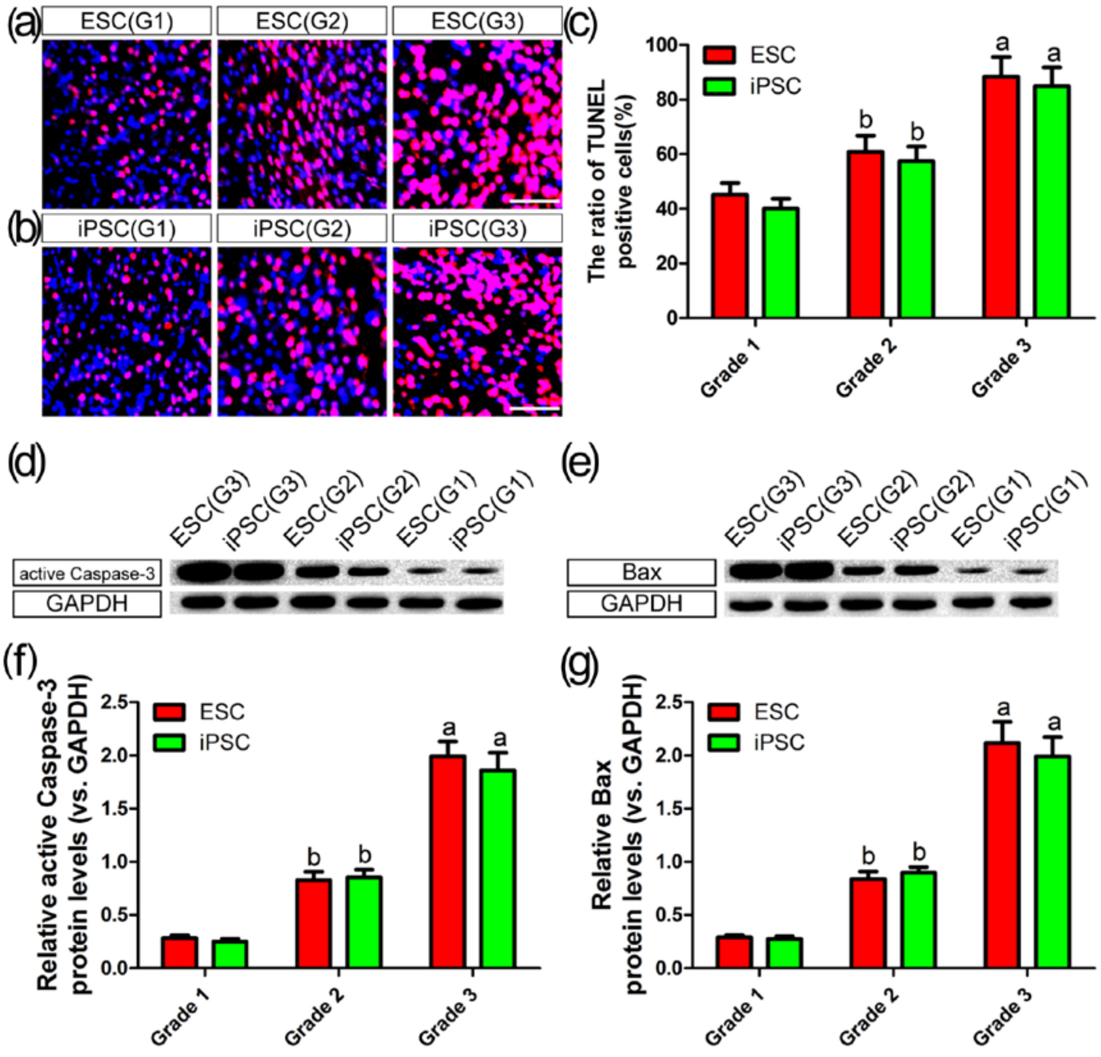

Supplementary Figure S4: TUNEL staining and Western blot. (a, b) Representative TUNEL-stained (red) and DAPI-stained (blue) brain section of mice subjected to syngeneic ESC grafts (a) or iPSC grafts (b) on day 14 post-implantation. (c) Histograms showed changes in the ratio of TUNEL positive cells in the brain of mice subjected to syngeneic ESC or iPSC grafts (n=3/group; a: P<0.05 versus grade 2 or grade 1; b: P<0.05 versus grade 1). (d, e) Representative immunoblots depicted the levels of active Caspase-3 (d) and Bax (e) in the brain. (f, g) Histograms showed that the levels of Caspase-3 (d) and Bax (e) in the brain (n=3/group; a: P<0.05 versus grade 2 or grade 1; b: P<0.05 versus grade 1). Scale bar=100 μm.

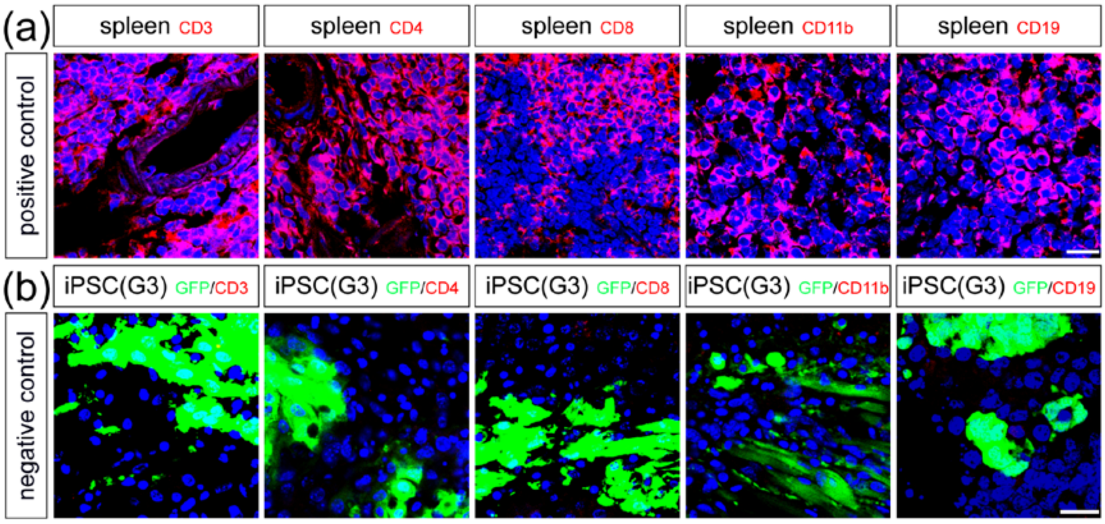

Supplementary Figure S5: Immunofluorescence. (a) Representative CD3-stained (red), CD4-stained (red), CD8-stained (red), CD11b-stained (red) or CD19-stained (red) and DAPI-stained (blue) spleen section of mice was used as positive control. (b) The omission of primary antibody in the brain section of mice subjected to syngeneic iPSC grafts (green) on day 14 post-implantation was used as negative control. Scale bar=25 μm.

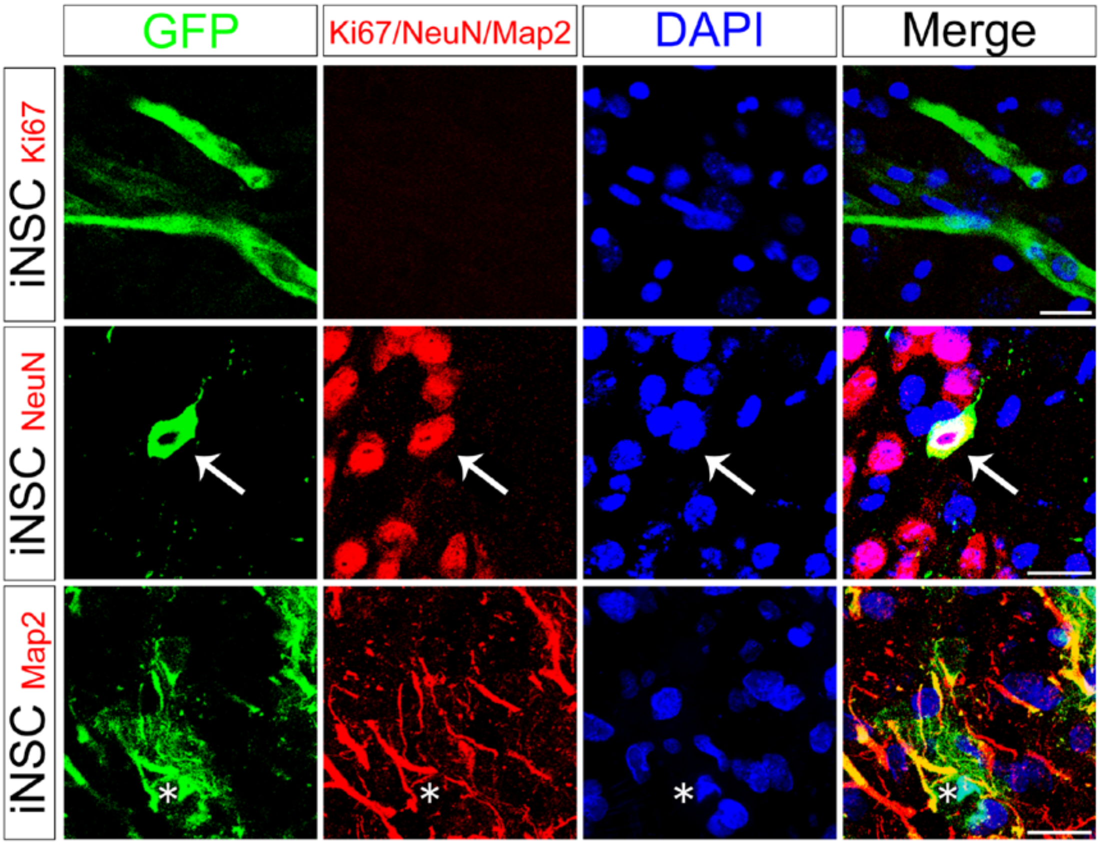

Supplementary Figure S6: Immunofluorescence. Immunofluorescence for NeuN (red) and Map2 (red) depicted that the GFP-expressing (green) iNSCs gave rise to neurons (arrow) and exhibited neuronal phenotype (asterisk), whereas these iNSC-derived cells (green) fail to express the cell-cycle marker Ki67 (red) 24 weeks after transplantation into the brain. Scale bar=10 μm.
